# Supplementary material for: The causal relationship between the human gut microbiota and pyogenic arthritis: a Mendelian randomization study
Source: Front Cell Infect Microbiol. 2024 Nov 26;14:1452480. doi: 10.3389/fcimb.2024.1452480 (PMC11629706; doi:10.3389/fcimb.2024.1452480)
Supplement: Supplementary file 1 [file DataSheet1.zip › mendelian test/ebi-a-GCST90027754.csv_forest.pdf]

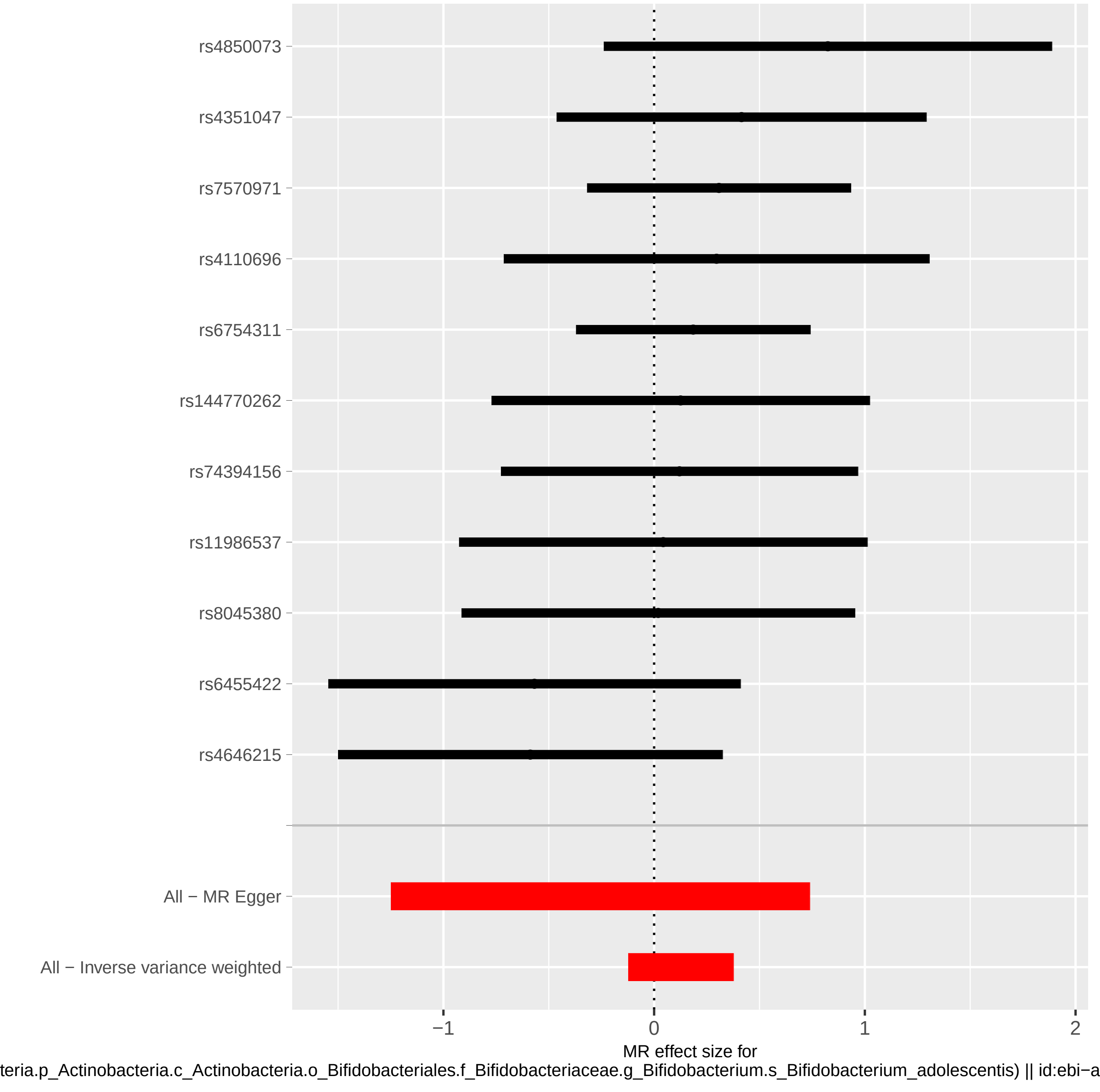
rs4850073
rs4351047
rs7570971
rs4110696
rs6754311
rs144770262
rs74394156
rs11986537
rs8045380
rs6455422
rs4646215
All − MR Egger
All − Inverse variance weighted
−1
0
1
2
MR effect size for
teria.p_Actinobacteria.c_Actinobacteria.o_Bifidobacteriales.f_Bifidobacteriaceae.g_Bifidobacterium.s_Bifidobacterium_adolescentis) || id:ebi−a
